# No Histological Difference between Large Atherosclerotic and Cardiogenic Embolic Thrombus

**DOI:** 10.1155/2022/4845264

**Published:** 2022-03-04

**Authors:** Rui Chen, Xiaopeng Zeng, Yong Liu, Cheng Huang, Jun Yang

**Affiliations:** ^1^Department of Neurology, The First Affiliated Hospital of Chongqing Medical University, Chongqing 400016, China; ^2^Department of Neurology, Chongqing University Three Gores Hospital, Chongqing 404100, China

## Abstract

**Background:**

The continuous development of endovascular treatment technology provides more opportunities for the histological study of thrombus. According to Trial of Org 10 172 in Acute Stroke Treatment (TOAST), clinicians take different strategies in anticoagulant or antiplatelet therapy. There are some patients still suffering from recurrent stroke while they took anticoagulant or antiplatelet drugs regularly for secondary prevention. In view of that, we found that histological analysis of thrombus can provide guidance for secondary prevention.

**Aim:**

Exploring the histological characteristics differences between large atherosclerotic and cardiogenic embolic thrombosis in order to guide clinical secondary prevention of the two stroke subtypes.

**Methods:**

A total of 54 patients with acute ischemic stroke were collected from December 2019 to April 2021. Identify stroke subtypes according to TOAST classification. Stain thrombus specimens with hematoxylin-eosin staining, and perform statistical analysis on the components (red blood cells and fibrin/platelets) of thrombus.

**Results:**

In cardiogenic thrombi, the composition of RBCs was dominant (51.38 ± 18.463%) compared to that of fibrin/platelets (48.62 ± 18.463%). Similarly, among the thrombi of large artery atherosclerotic, RBCs (50.40 ± 20.100%) compared to fibrin/platelets (49.60 ± 20.100%). There was no statistical difference in RBCs or fibrin/platelet composition of both cardiogenic and atherosclerotic thrombi (*P* = 0.89).

**Conclusions:**

The histologic composition of thrombi in cardiogenic and atherosclerotic had no statistical difference. These thrombi are all mixed thrombus, which are rich in RBCs, fibrinogen, and platelets. Anticoagulation combined with antiplatelet may be a more effective secondary prevention strategy.

## 1. Introduction

Acute ischemic stroke (AIS) has a high morbidity and disability rate. Endovascular treatment for AIS mainly includes mechanical thrombectomy and intravenous thrombolysis. Within the time window, endovascular treatments are recommended for patients with large vessel occlusion when ischemic penumbra was showed in imaging. Mechanical thrombus removal is currently one of the most effective treatment methods for acute large vessel occlusion. The continuous development of mechanical thrombectomy technology has promoted the pathological and histological researches of thrombus [1]. At present, the data on the success rate of intravenous thrombolysis is temporarily limited. Large clinical studies have shown that 41% of patients benefited from intravenous thrombolysis with rt-PA within 3 months [2]. The current study shows that intravenous thrombolysis is effective for anterior circulation occlusion, and about 51% of patients with basilar artery occlusion achieve recanalization [3].

For the analysis of the thrombus retrieved from endovascular treatments, some researches have shown that the components and structure of the thrombus are related to stroke subtypes [4–6]. But there are still differences or even contradictions between the current research results. The analysis of the components of thrombus can provide reference for the secondary prevention of clinical ischemic stroke [7]. The imaging manifestations of clots are various because of different compositions [8]. High-density blood clots tend to show high density of the middle cerebral artery on unenhanced CT, while the density of the blood clot is related to the level of hematocrit [9, 10]. According to Trial of Org 10 172 in Acute Stroke Treatment, there are differences in the choice of anticoagulant or antiplatelet drugs for secondary prevention among different etiologies of strokes. Studies have found that some patients still suffered from recurrent strokes after regular secondary prevention [11].

## 2. Aims

We analyze the histological characteristics of large atherosclerotic and cardiogenic embolic thrombus to provide a more accurate basis and reference for the secondary prevention of these two subtypes of stroke.

## 3. Methods

### 3.1. Study Design

Research included 54 patients who received mechanical thrombectomy in the Department of Neurology, the First Affiliated Hospital of Chongqing Medical University and Chongqing University Three Gorges Hospital, due to acute ischemic stroke from December 2019 to April 2021. The patients enrolled in the study need to meet the following conditions: (1) age over 18 years; (2) combined with risk factors for stroke, sudden focal neurological deficit, onset time less than 6 h; (3) National Institutes of Health Stroke Scale (NIHSS) score > 6 on admission; and (4) CTA or MRA confirming the occlusion of the large vessels including anterior intracranial circulation (internal carotid artery, middle cerebral artery M1-M2 segment, and anterior cerebral artery) and the posterior circulation (basilar artery) [12]. In addition, patients with any of the following points should be excluded: (1) CT or MRI showing extensive ischemic stroke (more than 1/3 of the cerebral hemisphere) and (2) history of spontaneous subarachnoid hemorrhage, intracranial artery malformation, or tumors.

The clots collected in the study are mainly from the internal carotid artery, middle cerebral artery, and basilar artery. Experienced neurologists classify the subtypes of stroke according to TOAST classification. The Ethics Committee of the First Affiliated Hospital of Chongqing Medical University approved the study [scientific research ethics (2020-017)]. The family members of each patient agreed to undergo endovascular treatment and perform histopathological analysis of the removed thrombus.

### 3.2. Processing of Clots

**Figure 1:**
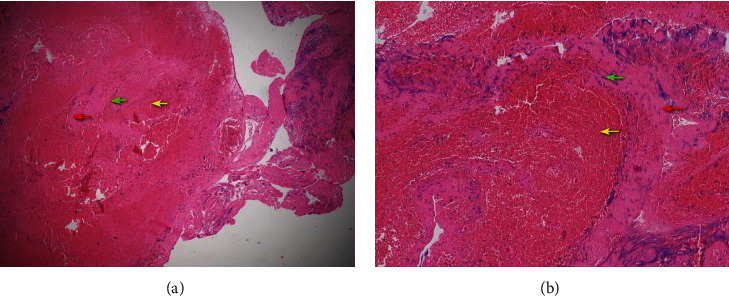
(a) A cardiogenic thrombus retrieved from a 73-year-old man with the occlusion of right internal carotid artery. (b) An atherosclerotic thrombus of a 72-year-old man with the occlusion of both right internal carotid artery and right middle cerebral artery. Between the two pictures, we can see the platelet trabecula (red arrow) and neutrophils (green arrow) are distributed around the platelet trabecula, and there were a large number of red blood cells (yellow arrow) between the trabeculae.

Use 10% neutral formalin for fixation for 24 hours, and then use 80%, 90%, 95%, and 100% ethanol for dehydration for 2 hours. After embedding in paraffin, paraffin sections with a thickness of 4-6 *μ*m are made. Finally, these samples were stained with Hematoxylin-Eosin. Olympus CX23 microscope was used to photograph these sections with an attached digital camera. Through H&E staining, red blood cells appeared red (Figure 1, yellow arrows), and platelets/fibrin appeared light red (Figure 1, red arrows). This experiment adopted a single-blind method. A pathologist who does not know the clinical diagnosis of the patient counted the areas of different stained areas under a microscope, so as to obtain the content of red blood cells and platelets/fibrin.

### 3.3. Statistical Analysis

SPSS software (version 26.0; IBM, Armonk, New York) was used for the statistical analysis. The Kolmogorov-Smirnov test was used to determine whether the proportion of red blood cells and fibrin/platelets in the thrombus is normally distributed. And two independent sample *t*-tests were used to evaluate whether there are differences in red blood cell or fibrin/platelet composition in different subtypes of stroke. *P* < 0.05 indicated that there was a significant statistical difference between two groups.

## 4. Results

The study recruited 54 patients, including 29 males and 25 females. According to TOAST classification, all patients were divided into large aortic atherosclerosis (LAA) group and cardiogenic embolization (CE) group.  Table 1 shows the basic data characteristics of the two groups of patients. The univariate analysis results show that the difference in NHISS score at admission and occlusion position of the two groups were statistically significant (*P* < 0.05). The results showed that there was no significant difference between the two groups in factors such as hypertension, diabetes, and smoking history. There was a significant difference between the two groups in the presence or absence of atrial fibrillation (*P* < 0.001).

Under the microscope, platelet trabeculae can be seen in the thrombus, and more red blood cells are trapped between the trabeculae (Figure 1). Microscopic observation suggests that cardiogenic and atherosclerotic thrombi were all mixed thrombus.

This study did not count the neutrophil composition, the following data were the RBCs and fibrin/platelet composition out of neutrophils. In cardiogenic thrombi, the composition of RBCs was dominant (51.38 ± 18.463%) compared to that of fibrin/platelets (48.62 ± 18.463%). Similarly, among the thrombi of large artery atherosclerotic, RBCs was also rich (50.40 ± 20.100%) compared to fibrin/platelets (49.60 ± 20.100%) (Table 2). There was no statistical difference in RBC composition of both cardiogenic and atherosclerotic thrombi (*P* = 0.89); also, there was no statistical difference in fibrin/platelet composition in the thrombi between the two subtypes of stroke either (Table 3).

## 5. Discussion

The study is aimed at finding the distinguished characters between cardiogenic and atherosclerotic thrombi, which can provide basis for secondary prevention of stroke. This study indicated that there were no statistical differences in RBCs or fibrin/platelets between both of them. This is contradictory to some current research results. Traditionally, platelets are mainly involved in thrombosis in the arterial system. The thrombus formed in the venous system is rich in fibrin and retained red blood cells. Ahn et al. studied 36 thrombus samples. The results showed that arterial thrombi had the highest red blood cell content, followed by fibrin, platelets, and white blood cells. Cardiac thrombosis has the highest fibrin content, followed by RBCs, platelets, and white blood cells [6]. Marder et al. conducted the study of 25 cases of acute ischemic stroke with mechanical removal of thrombi in the MCA and ICA; histological analysis showed that there was no significant difference in the content of thrombus in cardiogenic stroke and large atherosclerotic stroke [13].

The current strategies for preventing stroke recurrence mainly include lifestyle modification and intervention, antiplatelet, anticoagulation, controlling hypertension, controlling diabetes, controlling, and the level of lipids [14]. Studies have shown that the use of antiplatelet therapy within 48 hours of 40,000 ischemic strokes for 1-2 months reduces the incidence of recurrence of ischemic strokes by 23% [15]. Another study suggests that in 23748 LAA patients, anticoagulation started within 48 hours of onset increases the risk of intracranial hemorrhage but does not reduce the incidence of stroke recurrence [16]. Anticoagulation was started within 48 hours after the onset of cardiogenic stroke, and it did not significantly reduce the recurrence of early ischemic stroke, and symptomatic intracranial hemorrhage increased [17]. In stroke with atrial fibrillation, warfarin can reduce the occurrence of stroke, and it is currently the gold standard for the prevention of primary and secondary stroke in patients with atrial fibrillation [18]. In large randomized clinical trials, the new oral anticoagulants (NOACs) are similar to warfarin in preventing ischemic stroke, and the risk of intracranial hemorrhage (ICH) is relatively low [19]. In another study, 574 patients with atrial fibrillation were followed up for an average of 4.9 years and found that antiplatelet combined with anticoagulation was superior to anticoagulation alone [20]. A meta-study showed no increased risk of minor or nonserver bleeding with anticoagulation combined with antiplatelet therapy compared with anticoagulation alone [21]. Systemic atherosclerosis often causes peripheral arterial disease, which in turn leads to cardiovascular disease and limb loss. Antiplatelet therapy has been the mainstay of treatment in the past few years, but the incidence of cardiovascular events remains high. Studies have shown that new oral anticoagulants combined with aspirin can improve the patient benefit rate [22]. Therapeutic doses of warfarin reduce recurrent ischemic events after myocardial infarction but also increase the risk of bleeding [23].

According to statistics, recurrent strokes account for 25-30% of all strokes [24]. Effective and appropriate secondary prevention strategies can greatly reduce the incidence of stroke recurrence. After regular antiplatelet or anticoagulant therapy, some stroke patients still have recurrent strokes. Based on our findings in this research, since both LAA and CE thrombus are mixed thrombus, there is no statistical difference in platelet/fibrin and RBCs content. We propose the hypothesis that antiplatelet and anticoagulation treatments are used simultaneously for the two subtypes of strokes, which may reduce the incidence of recurrent strokes. But at the same time, it may increase the risk of intracranial hemorrhage. Using of antiplatelet and anticoagulant drugs with a relatively low risk of intracranial hemorrhage can help reduce the incidence of intracranial hemorrhage. And further clinical research is needed to reduce intracranial hemorrhage.

## 6. Conclusion

In this study, we studied 54 thrombus samples to explore the difference in composition. And we found that there was no difference in RBCs or fibrin/platelets content of LAA and CE thrombus. Recurrent strokes account for a significant portion of strokes; effective secondary prevention strategies can reduce the risk of recurrent stroke. Based on the findings of this study, we believe that anticoagulation combined with antiplatelet can reduce the recurrence of stroke.

## Figures and Tables

**Table 1:** Clinical characteristics of the 54 patients recruited in this study.

Variables		Number	LAA group (25)	CE group (29)	*P* value
Age			63.7 ± 12.1	73.4 ± 9.1	0.131
Sex	Male	29	16	13	
	Female	25	9	16	
NIHSS on admission, median (IQR)			13 (9-21)	15 (10-18)	0.014
Hypertension	Yes	33	16	17	0.686
	No	21	9	12	
Diabetes	Yes	9	4	5	0.903
	No	45	21	24	
Atrial fibrillation	Yes	27	2	25	<0.001
	No	27	23	4	
Smoking	Yes	23	13	10	0.194
	No	31	12	19	
Occlusion site	ICA or MCA	50	23	27	0.887
	BA or PCA	4	2	2	

ICA: internal carotid artery; MCA: middle cerebral artery; BA: basilar artery PCA: posterior cerebral artery.

**Table:** (a) Fibrin/platelets

	*N*	Minimum	Maximum	Mean	Std. deviation
*F*/*P*	29	20	90	48.62	18.463
Valid *N*	29				

**Table:** (b) RBC

	*N*	Minimum	Maximum	Mean	Std. deviation
RBC	29	10	80	51.38	18.463
Valid *N*	29				

**Table:** (c) Fibrin/platelets

	*N*	Minimum	Maximum	Mean	Std. deviation
*F*/*P*	25	10	90	49.60	20.100
Valid *N*	25				

**Table:** (d) RBC

	*N*	Minimum	Maximum	Mean	Std. deviation
RBC	25	10	90	50.40	20.100
Valid *N*	25				

**(a):** 

		*F*	Sig	*t*	Df	Sig (2-tailed)	Mean difference	Etd. error difference	95% CI
RBC	Equal variances assumed	.019	.890	-.187	52	.853	-.979	5.250	-11.514~9.555
	Equal variances not assumed			-.185	49.269	.854	-.979	5.283	-11.595~9.637

**(b):** 

		*F*	Sig	*t*	Df	Sig (2-tailed)	Mean difference	Etd. error difference	95% CI
*F*/*P*	Equal variances assumed	.019	.890	-.187	52	.853	-.979	5.250	-9.555~11.514
	Equal variances not assumed			-.185	49.269	.854	-.979	5.283	-9.637~11.595

## Data Availability

The data used to support the findings of this study are available from the corresponding author upon request.
